# COVID-19 Immunization Coverage Among People With Sickle Cell Disease

**DOI:** 10.1001/jamanetworkopen.2023.51618

**Published:** 2024-01-08

**Authors:** Hannah K. Peng, Kevin J. Dombkowski, Melissa A. Plegue, Krista Latta, Ryan Malosh, Melissa S. Creary, Sarah L. Reeves

**Affiliations:** 1 Susan B. Meister Child Health Evaluation and Research (CHEAR) Center, University of Michigan, Ann Arbor; 2 Michigan Department of Health and Human Services, Lansing; 3 School of Public Health, University of Michigan, Ann Arbor

## Abstract

This cross-sectional study compares the completion of the primary COVID-19 vaccine series in Michigan residents with vs without sickle cell disease and by age group.

## Introduction

In the US, people living with sickle cell disease (SCD) are at high risk for hospitalization and mortality from COVID-19.^1^ Although COVID-19 immunization reduces the risk of severe illness, the few studies of vaccination among the SCD population are limited to health systems.^1,2^ We assessed COVID-19 immunization coverage among Michigan residents with SCD compared with residents without SCD.

## Methods

COVID-19 vaccination records (as of August 1, 2022) for residents 5 years or older were obtained from the Michigan Care Improvement Registry (MCIR). Individuals with SCD were identified through linkage to the Michigan Sickle Cell Data Collection (MiSCDC) program; all other residents were classified as without SCD. The University of Michigan Institutional Review Board determined this project to be not regulated as it is public health surveillance. We followed the STROBE reporting guideline.

Study outcome was COVID-19 primary immunization series completion. We calculated the proportions of residents with and without SCD who completed at least 1 dose of Ad26.COV2.S (Janssen) or 2 doses of mRNA-1273 (Moderna) or BNT162b2 (Pfizer/BioNTech), or other COVID-19 vaccine overall and by age group. Denominators came from MiSCDC (with SCD group) and 2019 US Census (without SCD group). Race and ethnicity data are underreported in the MCIR and therefore were not included in the present analyses.

We used SAS 9.4 (SAS Institute Inc) to compare proportions with relative risk (RR) and Breslow-Day tests; 1-sided *P* < .05 was considered significant. Additional methodological details are provided in the eMethods in Supplement 1.

## Results

There were 3424 people with SCD (1997 females [58.3%], 1427 males [41.7%]; 2489 [72.7%] aged 18-64 years) and 9 416 991 without SCD (4 789 163 females [50.9%], 4 627 828 males [49.1%]; 6 075 034 [64.5%] aged 18-64 years) in the study population (Table 1). COVID-19 immunization coverage for people with SCD was 33.5%, nearly half of the 61.3% coverage for people without SCD (RR, 0.55; 95% CI, 0.52-0.57) (Table 2).

**Table 1.** Demographic Distribution of Study Population

Characteristic	Study population, No. (%)	
	With SCD (n = 3424)	Without SCD (n = 9 416 991)
Age group, y^a^		
5-11	454 (13.3)	824 959 (8.8)
12-17	371 (10.8)	751 707 (8.0)
18-64	2489 (72.7)	6 075 034 (64.5)
≥65	110 (3.2)	1 765 291 (18.7)
Sex		
Female	1997 (58.3)	4 789 163 (50.9)
Male	1427 (41.7)	4 627 828 (49.1)

Abbreviation: SCD, sickle cell disease.

^a^ As of July 1, 2022.

**Table 2.** COVID-19 Immunization Completion Coverage by Sickle Cell Disease (SCD) Status

Group	% of Population with completed immunization (95% CI)		Relative risk (95% CI)
	With SCD (n = 3424)	Without SCD (n = 9 416 991)	
Overall	33.5 (31.9-35.1)	61.3 (61.3-61.3)	0.55 (0.52-0.57)
Age group, y^a^			
5-11	16.7 (13.4-20.5)	24.6 (24.5-24.7)	0.68 (0.55-0.84)
12-17	31.0 (26.3-36.0)	41.3 (41.2-41.4)	0.75 (0.64-0.87)
18-64	35.2 (33.3-37.1)	61.2 (61.1-61.2)	0.58 (0.55-0.61)
≥65	73.6 (64.4-81.6)	87.4 (87.3-87.4)	0.84 (0.75-0.94)

Abbreviation: SCD, sickle cell disease.

^a^ Breslow-Day *P* < .001 across age groups.

Immunization completion increased with age for people with SCD (range, 17%-74%) and without SCD (range, 25%-87%). Relative risks by age group were 0.68 (95% CI, 0.55-0.84) for 5 to 11 years, 0.75 (95% CI, 0.64-0.87) for 12 to 17 years, 0.58 (95% CI, 0.55-0.61) for 18 to 64 years, and 0.84 (95% CI, 0.75-0.94) for 65 years or older. The association between completion and SCD status (with vs without) was significantly different across age groups (RR, 0.68 vs 0.75 vs 0.58 vs 0.84; *P* < .001).

## Discussion

In this population-level study, COVID-19 immunization completion was nearly 2 times lower for people with vs without SCD. This discrepancy was particularly large among adults aged 18 to 64 years. These results are consistent with findings from health system–based studies in which fewer than half of patients with SCD completed the vaccination series.^2^ Misinformation and mistrust are reasons frequently associated with low COVID-19 immunization completion in the SCD community,^3,4^ which are also common throughout the Black population in the US.^5,6^

This study has several limitations. First, it began shortly after approval of vaccines for those aged 5 to 12 years, limiting time for immunization uptake in this group. Second, we did not consider immunization scheduling rules (eg, minimum intervals), which may have led to overestimation of immunization coverage across the study population. Third, MiSCDC uses multiple data sources to identify SCD cases with validated methods. However, individuals with SCD born outside Michigan or not using health services were excluded, which may have led to overestimated immunization coverage for the SCD population. Fourth, although the study was performed in a racially and ethnically diverse state with average SCD representation, the results may not be generalizable to states with different COVID-19–related policies and perceptions.

Future research should include perspectives regarding COVID-19 vaccines among individuals with SCD and their caregivers. Results of such studies may inform targeted interventions to increase immunization coverage and reduce the risk of COVID-19–associated morbidity and mortality in this high-risk population.

## Notes

Supplement 1.
eMethods.

eReferences

Supplement 2.
Data Sharing Statement
