# Supplementary material for: COVID-19 Immunization Coverage Among People With Sickle Cell Disease
Source: JAMA Netw Open. 2024 Jan 8;7(1):e2351618. doi: 10.1001/jamanetworkopen.2023.51618 (PMC10774988; doi:10.1001/jamanetworkopen.2023.51618)
Supplement: Supplement 1. — eMethods. eReferences [file jamanetwopen-e2351618-s001.pdf]

Supplemental Online Content

Peng HK, Dombkowski KJ, Plegue MA, et al. COVID-19 immunization coverage among people with sickle cell disease. *JAMA Netw Open*. 2024;7(1):e2351618. doi:10.1001/jamanetworkopen.2023.51618

eMethods

eReferences

This supplemental material has been provided by the authors to give readers additional information about their work.

© 2024 Peng HK et al. *JAMA Network Open*

eMethods

Data sources were the Michigan Care Improvement Registry (MCIR), the Michigan Sickle Cell Data Collection (MiSCDC) program, and the 2019 US Census. MCIR is a lifespan immunization registry that documents immunizations given to Michigan residents. The Centers for Disease Control and Prevention (CDC) expect that all COVID-19 immunizations administered in Michigan to people of any age be entered into MCIR within 72 hours.[1] This makes MCIR the "gold standard" of COVID-19 immunization information in Michigan. MiSCDC is a public health surveillance system which leverages multiple secondary data sources, including newborn screening, Medicaid, clinic, and hospital records to identify the population of people with sickle cell disease (SCD) in Michigan.[2] Acquisition of data to conduct surveillance is made possible through designation of public health authority granted by the Michigan Department of Health and Human Services. Previous studies have indicated that MiSCDC captures twice as many people living with SCD than single databases alone (e.g. Medicaid; hospital discharge).[3] Combined, these datasets enable a population-level assessment of COVID-19 immunization among people with SCD in Michigan.

COVID-19 vaccine was approved under emergency use authorization on 12/18/2020 for adults 18+, 5/10/2021 for adolescents 12+, and 10/29/2021 for children 5+. COVID-19 immunization records were obtained (8/1/2022) for individuals aged ≥5 years on 7/1/2022 to allow a 1-month period for the youngest recipients to complete the immunization series. Doses were excluded for people flagged as deceased, for those who opted out of MCIR (<0.1% of all people in MCIR), for doses flagged as deleted, and for additional doses received in the same day by the same person. Using name, sex, and birthdate from MCIR, individuals with ≥1 COVID-19 immunization were linked to the MiSCDC using probabilistic linkage software (LinkageWiz).[4] Manual review of matched pairs was employed to maximize accuracy of the linked individuals. MiSCDC uses validated case definitions to identify individuals living with SCD.[5, 6] These case definitions include CLIA certified laboratory result of SCD by state NBS program with confirmatory testing, clinical diagnosis with documented confirmatory CLIA-certified laboratory testing after newborn period, and administrative claims-based criteria. All SCD genotypes were included in the study population. All individuals with COVID-19 immunization doses not linked to MiSCDC were considered non-SCD.

The study outcome was COVID-19 completion coverage, defined as primary series completion (≥1 dose for Janssen, ≥2 doses for all other manufacturers).[7] Date of COVID-19 immunization series completion was determined as the date of the first dose for Janssen or second dose for all other manufacturers. The completion coverage denominator for SCD included all people who met validated case definitions for SCD in MiSCDC, age 5+, with evidence of Michigan residency during the preceding 5 years (e.g., received healthcare in Michigan, enrolled in Michigan Medicaid). The denominator for people without SCD was based on the 2019 Michigan population (US Census), minus the number of those aged <5 and the number of people in the SCD denominator. Completion coverage was calculated as the proportion of people who had completed the primary COVID-19 immunization series within each group (SCD and non-SCD), both overall and by age group (5-11 years, 12-17 years, 18-64 years, and 65+ years). These age groups were chosen based on the groups for which the COVID-19 vaccine was approved by emergency use authorizations over time. SCD and non-SCD completion coverage were compared within age groups using relative risk and across age groups using a Breslow Day test.

© 2024 Peng HK et al. *JAMA Network Open*

eReferences

1. CDC. Data Definitions for COVID-19 Vaccinations in the United States. Updated October 13, 2023. Accessed November 16, 2023, https://www.cdc.gov/coronavirus/2019-ncov/vaccines/reporting-vaccinations.html
2. Michigan Sickle Cell Data Collection. MiSCDC Program. Updated 2023. Accessed November 16, 2023, https://www.miscdc.org/
3. Reeves SL, Horiuchi S, Zhou M, et al. Case Ascertainment of Sickle Cell Disease Using Surveillance or Single Administrative Database Case Definitions. *Public health reports (Washington, DC : 1974)*. May 19 2023:333549231166465. doi:10.1177/00333549231166465
4. LinkageWiz. LinkageWiz Data Matching Software. Accessed November 16, 2023, https://linkagewiz.net/
5. Snyder AB, Zhou M, Theodore R, Quarmyne MO, Eckman J, Lane PA. Improving an Administrative Case Definition for Longitudinal Surveillance of Sickle Cell Disease. *Public Health Rep*. May/Jun 2019;134(3):274-281. doi:10.1177/0033354919839072
6. Reeves S, Garcia E, Kleyn M, et al. Identifying Sickle Cell Disease Cases Using Administrative Claims. *Acad Pediatr*. 2014;14(5 Suppl):S61-S67. doi:10.1016/j.acap.2014.02.008
7. Michigan Department of Health & Human Services. COVID-19 Vaccine Dashboard. Updated November 8, 2023. Accessed November 16, 2023, https://www.michigan.gov/coronavirus/resources/covid-19-vaccine/covid-19-dashboard

© 2024 Peng HK et al. *JAMA Network Open*
