# Supplementary material for: COVID-19 Immunization Coverage Among People With Sickle Cell Disease
Source: JAMA Netw Open. 2024 Jan 8;7(1):e2351618. doi: 10.1001/jamanetworkopen.2023.51618 (PMC10774988; doi:10.1001/jamanetworkopen.2023.51618)
Supplement: Supplement 2. — Data Sharing Statement [file jamanetwopen-e2351618-s002.pdf]

Data Sharing Statement

Peng. COVID-19 Immunization Coverage Among People With Sickle Cell Disease. *JAMA Netw Open*. Published January 08, 2024. doi:10.1001/jamanetworkopen.2023.51618

Data
Data available: No
